# TGFβ1 reinforces arterial aging in the vascular smooth muscle cell through a long-range regulation of the cytoskeletal stiffness

**DOI:** 10.1038/s41598-018-20763-w

**Published:** 2018-02-08

**Authors:** Wanqu Zhu, Byoung Choul Kim, Mingyi Wang, Jessie Huang, Abraham Isak, Natalia M. Bexiga, Robert Monticone, Taekjip Ha, Edward G. Lakatta, Steven S. An

**Affiliations:** 1 Department of Environmental Health and Engineering, Johns Hopkins Bloomberg School of Public Health, Baltimore, MD 21205 USA; 2 Department of Biophysics and Biophysical Chemistry, Johns Hopkins University, Baltimore, MD 21205 USA; 3 Department of Biomedical Engineering, Johns Hopkins University, Baltimore, MD 21218 USA; 4 Howard Hughes Medical Institute, Baltimore, Maryland 21218 USA; 5 Division of Nano-Bioengineering, Incheon National University, Incheon, Republic of Korea; 6 Laboratory of Cardiovascular Science, Intramural Research Program, National Institute on Aging, National Institutes of Health, Baltimore, MD 21224 USA; 7 Immunobiological and Biopharmaceutical Laboratory, Department of Pharmaceutical Biochemistry Technology, University of Sao Paulo, Sao Paulo, Brazil; 8 Department of Chemical and Biomolecular Engineering, Johns Hopkins University, Baltimore, MD 21218 USA; 9 Department of Biomedical Engineering, Ulsan National Institute of Science and Technology, Ulsan, Republic of Korea

## Abstract

Here we report exquisitely distinct material properties of primary vascular smooth muscle (VSM) cells isolated from the thoracic aorta of adult (8 months) *vs*. aged (30 months) F344XBN rats. Individual VSM cells derived from the aged animals showed a tense internal network of the actin cytoskeleton (CSK), exhibiting increased stiffness (elastic) and frictional (loss) moduli than those derived from the adult animals over a wide frequency range of the imposed oscillatory deformation. This discrete mechanical response was long-lived in culture and persistent across a physiological range of matrix rigidity. Strikingly, the pro-fibrotic transforming growth factor β1 (TGFβ1) emerged as a specific modifier of age-associated VSM stiffening *in vitro*. TGFβ1 reinforced the mechanical phenotype of arterial aging in VSM cells on multiple time and length scales through clustering of mechanosensitive α_5_β_1_ and α_v_β_3_ integrins. Taken together, these studies identify a novel nodal point for the long-range regulation of VSM stiffness and serve as a proof-of-concept that the broad-based inhibition of TGFβ1 expression, or TGFβ1 signal transduction in VSM, may be a useful therapeutic approach to mitigate the pathologic progression of central arterial wall stiffening associated with aging.

## Introduction

Increased stiffness of central arteries as measured by pulse wave velocity (PWV) is a major risk factor for a broad spectrum of cardiovascular diseases (CVD)^1,2^, and is an independent predictor for CVD-associated morbidity and mortality in Western society^3^. Stiffening of these large elastic arteries is now construed as time-dependent^4^, and when considered with other covariates inherent in Western lifestyle, increases exponentially in human beyond the age of 40 years^5^. In the United States alone, it is estimated that 35 million individuals are older than 65 years^6^. As the average age of population projected to grow markedly in the coming decades, there is an unmet clinical need for understanding the nature of arterial aging, and developing new therapeutic modalities to prevent or treat the pathologic progression of age-associated arterial wall stiffening.

The Fisher 344, or, cross-bred Brown Norway (F344XBN) rats have emerged as a good animal model of arterial aging^7^, exhibiting age-related structural changes in the vascular wall that are grossly congruent with the *physiologic* changes in human lifetime^8–11^. For example, analogous to the ‘normal’ aging in humans, the aortic wall of aged F344XBN rats is marked by low-grade inflammation, the emergence of pro-fibrotic transforming growth factor β1 (TGFβ1), and thickening of the intima, media and adventitia layers^12,13^. Further, within these layers of the aortic wall, there is loss of elastin, gain of collagen and fibronectin^8,12,13^ as well as accumulation and infiltration of α-smooth-muscle-actin positive structural cell types^9,11,14^. Some of these structural changes have been also documented in other aging studies employing different experimental animals, from mice to non-human primates, and under physiologic and pathologic conditions^15–18^.

The current view stipulates that age-associated stiffening of the central arteries is largely attributable to changes in the structural composition and the elasticity of extracellular matrix (ECM) in the vascular wall that are driven by pro-inflammatory milieu and accentuated by pre-existing metabolic syndrome, diabetes and hypertension^19–21^. Contribution of vascular smooth muscle (VSM), the structural cell-types of the vascular wall, to arterial remodeling/stiffening is poorly understood. In addition, the molecular-level mechanical transgression in VSM cells forcing age-associated stiffening of central arteries is unknown.

Using F344XBN experimental model, here we explored the stiffness landscape of arterial aging in VSM, at the single-cell resolution, with magnetic twisting cytometry (MTC). Compared with primary VSM cells derived from adult rats (8 months), those derived from aged rats (30 months) exhibited increased stiffness deep within the cytoskeletal structures. The increase in cell stiffness was persistent in culture, prevailed under a wide variety of matrix rigidities, and positively associated with TGFβ1 expression and its receptor activation. Applying small-scale tension gauge tether (TGT) and large-scale Fourier transform traction microscopy (FTTM) methods, we further demonstrated that the mechanical phenotype of arterial aging in VSM cells is reinforced by TGFβ1 and is propagated, at long distance, through a cluster of mechanosensitive integrin receptors α_5_β_1_ and α_v_β_3_.

## Results and Discussion

### Cellular models of *physiologic* arterial aging *in vitro*

There is a widespread agreement that increased smooth muscle mass (i.e. hyperplasia and hypertrophy), as well as alterations in the amount and composition of ECM (i.e. collagen and fibronectin) are the major contributing factors for age-associated remodeling and stiffening of the arterial wall^22,23^. As reported previously^24^, compared with adult (8 months) F344XBN rats, aged (30 months) rats showed anatomically distinct structural remodeling in the central aorta and, in particular, thickening of the intima, media and adventitia layers (Suppl. Fig. 1a). Moreover, consistent with earlier studies^14,18^, primary VSM cells–as marked by the expression of smooth muscle myosin heavy chains and α-smooth-muscle actin (Suppl. Fig. 1b,c)–derived from the aged rats proliferated at a faster rate in culture than those derived from the respective aortic segments of adult rats (Suppl. Fig. 1d).

Using this phenotypically, and epigenetically, well-congruent cellular model of *physiologic* aging^8–11^, here we interrogated the physical state of these structural cell types of the central aorta applying a series of live cell micromechanical methods. Herein, we used ‘young’ *vs*. ‘old’ to describe individual VSM cells derived from the adult (8 months) *vs*. aged (30 months) rats. For these studies, we used isolated primary VSM cells that had been passaged in standard culture media and under identical experimental conditions.

### Age-associated changes in the material properties of VSM cells deep within the cytoskeletal network

First, we applied *spontaneous* motions of microbeads functionalized to the living cytoskeleton (CSK)^25–31^ and measured the rate of CSK remodeling in isolated primary VSM cells (Suppl. Fig. 2). In both young and old VSM cells, the computed mean square displacements (MSD) of *unforced* bead motions in 2D increased with time (*t*) as a power law with an exponent α greater than unity (~1.6; Suppl. Fig. 2b,c), indicating non-thermal, molecular-level fluctuations of the underlying CSK structures^32,33^. Such non-Brownian dynamics of the cytoskeletal network were unremarkably similar in both young and old VSM cells, though (Suppl. Fig. 2c,d).

**Figure 1:**
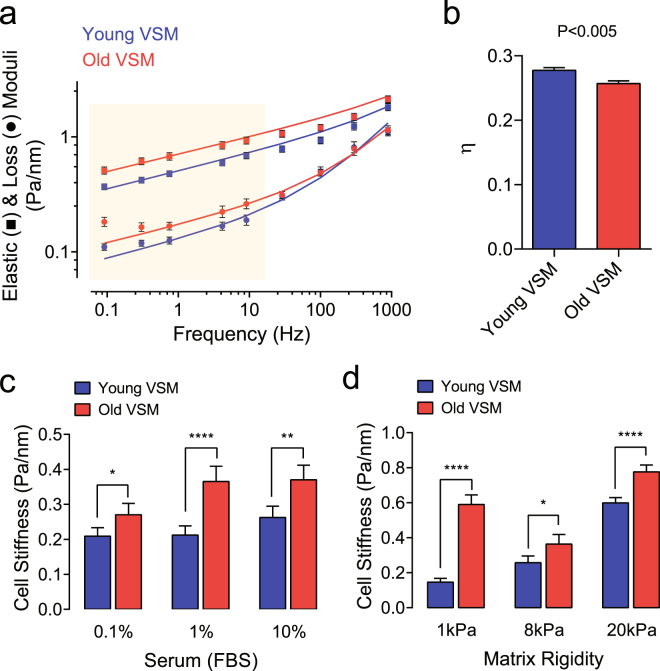
Material properties of young *vs*. old VSM cells as measured by MTC. **(a)** Using MTC, we measured cell stiffness (g’, storage modulus) and internal friction (g”, loss modulus) over 5 decades of probing frequency. Data are presented as Geometric Mean ± SE (young VSM, n = 466 cells; old VSM, n = 298 cells). The solid lines are the fit of experimental data to the structural damping equation with addition of a Newtonian viscous term as previously described^34^. A shaded box indicates statistical differences between young *vs*. old VSM cells. (**b**) Corresponding hysteresivity η (the ratio of g” to g’) detected at 0.75 Hz. Data are presented as Mean ± SE. **(c)** Stiffness of VSM cells adhered on collagen-coated plastic wells cultured in media containing a varying FBS concentration (0.1–10%). Data are presented as Geometric Mean ± SE (n = 113–195 individual cell measurements for each condition). **(d)** Stiffness of VSM cells (1% FBS) cultured on collagen-coated elastic gels with varying rigidity (1–20 kPa with Poisson’s ratio of 0.48). Data are presented as Geometric Mean ± SE (1 kPa, n = 175–370 individual cell measurements; 8 kPa, n = 68–119 individual cell measurements; 20 kPa, n = 401–520 individual cell measurements). *P < 0.05; **P < 0.01; ****P < 0.0001. Herein, we report cell stiffness measured at 0.75 Hz. For measured stiffness and friction over 5 decades of probing frequency, please see Supplementary Figure 3.

In order to probe deeper into the mechanical properties of the underlying cytoskeletal structures, and the molecular relaxation processes that might be associated with aging, we next applied *forced* motions of the same functionalized beads with magnetic twisting cytometry (MTC)^26,31,34^. For each individual VSM (young *vs*. old), we computed the elastic modulus g’ (stiffness), the loss or viscous modulus g” (internal friction), and the loss tangent η (the ratio of g”/g’) over a wide frequency range. Throughout the measurement range (oscillatory frequencies from 10^−1^ to 10^3^ Hz), the stiffness of VSM cells (young and old) increased with frequency (*f*) as a weak power law (Fig. 1a). The internal friction or energy dissipation also followed a weak power law at low frequencies (below ~10 Hz), with nearly the same exponent as did the stiffness of young (*f* ^0.154^) and old (*f* ^0.149^) VSM cells (Fig. 1a). At higher frequencies (above ~10 Hz), however, the dissipative response showed a progressively stronger frequency dependence, approaching but never quite attaining a power-law exponent of 1–i.e. characteristic of a Newtonian viscosity^34^.

Accordingly, consistent with the original work by Fabry *et al*.^34^, over a five decade of frequency, molecular dynamics of the cytoskeletal network within the VSM derived from the classically elastic arteries were also distributed broadly. There were many relaxation processes contributing when the frequency of the imposed deformation was small, but very few as the frequency was increased and slower processes became progressively frozen out of the response. This class of single-cell rheological response is empirically in line with the mechanical behavior of cell-ensemble in living tissues^35^, and is equivalent to the macroscopic rheological properties of arterial wall^2,36^.

As shown in Fig. 1a,b, however, the power-law frequency dependence of stiffness (g’) and friction (g”) differed appreciably between young *vs*. old VSM cells, including the loss tangent η and the derived parameter ‘x’–the so called an ‘effective temperature’ of the cytoskeletal matrix (young VSM, x = 1.174 ± 0.003 *vs*. old VSM, x = 1.162 ± 0.003; Mean ± SE, n = 298–466 individual cell measurements; P < 0.005)^34^. For example, if g’(*f*) goes as ~*f* ^x−1^, x represents effective matrix temperature and determines where the cells sit along a continuous spectrum of solid-like (x = 1) *vs*. liquid-like (x = 2) states^26,30,34^. In the limit that x approaches 1, the behavior approaches that of a Hookean elastic solid and, in the limit in which x approaches 2, the behavior approaches that of a Newtonian viscous fluid. Hence, purely as a matter of phenomenology, individual VSM cells derived from aged rats are in a physical state that is more solid-like and closer to a glass transition than those derived from adult rats^26,31,34^.

Most strikingly, these material behaviors of isolated primary VSM cells and, in particular, the increased cytoskeletal stiffness in old VSM cells was persistent in culture (Fig. 1c) and prevailed under a wide range of matrix rigidities in the physiologic spectrum of arterial wall stiffness (Fig. 1d and Suppl. Fig. 3). These results indicate a long retention/memory of material stiffness in VSM cells that is hardwired, and perhaps epigenetically regulated, to arterial aging.

### Phenotypic expression of increased cytoskeletal stiffness is linked to canonical TGFβ1 signal transduction in VSM cells

**Figure 2:**
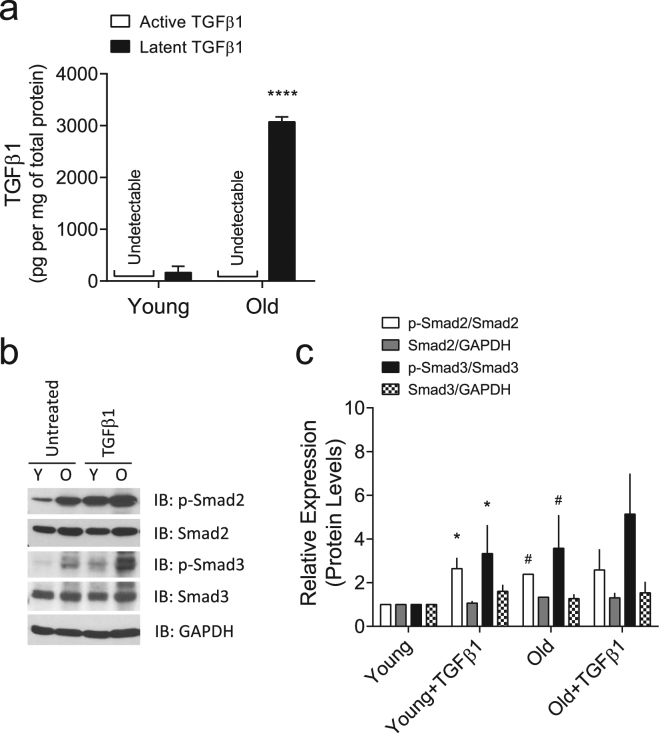
TGFβ1 expression and signaling in VSM cells. (**a**) Production of TGFβ1 by young *vs*. old VSM cells as detected by sandwich enzyme-linked immunosorbent assay. Data are presented as Mean ± SD (n = 3). (**b**,**c**) VSM cells (young and old) were treated for 24 h with or without 5 ng/ml TGFβ1. (**b**) Phosphorylation levels of Smad2/3 were detected by western blot (Full gel/blot is shown in the Supplementary Figure 5). (**c**) Quantitation of the protein levels. Data are expressed as Mean ± SD (n = 3). *P < 0.05 (untreated *vs*. TGFβ1 treated); ^#^*P* < 0.05 (young *vs*. old).

In aged rats, we had identified an altered pro-fibrotic TGFβ1 expression within the α-smooth-muscle actin-rich compartment of the stiffened aortic wall^13^. Consistent with these results, primary VSM cells isolated from aged rats retained *in vitro* higher expression levels of latent TGFβ1 than those derived from adult rats (Fig. 2a). At baseline condition, the phosphorylation levels of Smad2/3 were also higher in old VSM cells (Fig. 2b,c), suggesting an inherent activation of the TGFβ1 receptor in old VSM cells. Of note, exogenous addition of TGFβ1 increased the phosphorylation levels of Smad2/3 in both young and old VSM cells; however, the increase was more robust in young VSM cells (Fig. 2b,c).

**Figure 3:**
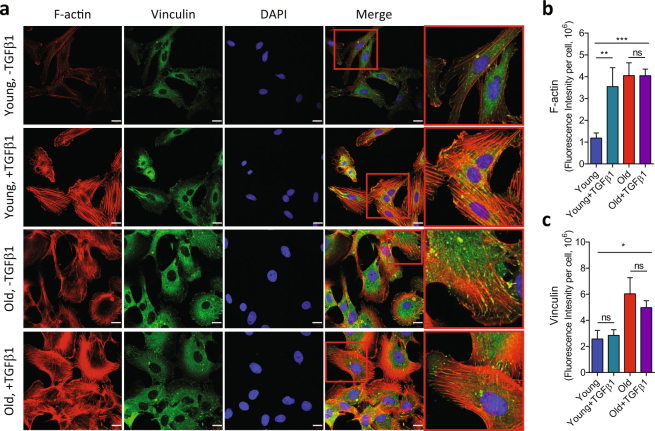
Immunofluorescent detection of actin cytoskeletal structures in VSM cells. VSM cells (young and old) were treated for 24 h with or without 5 ng/ml TGFβ1, and the internal cytoskeletal structures were visualized with actin cytoskeleton/focal adhesion staining kit as described in the Methods (**a**). Scale bar is 20 µm. Average fluorescent intensities of (**b**) phalloidin and (**c**) vinculin per cell from multiple images are presented as Mean ± SE (Young: n = 7; Young + TGFβ1: n = 5; Old: n = 7; Old + TGFβ1: n = 6). *P < 0.05; **P < 0.01; ***P < 0.001.

**Figure 4:**
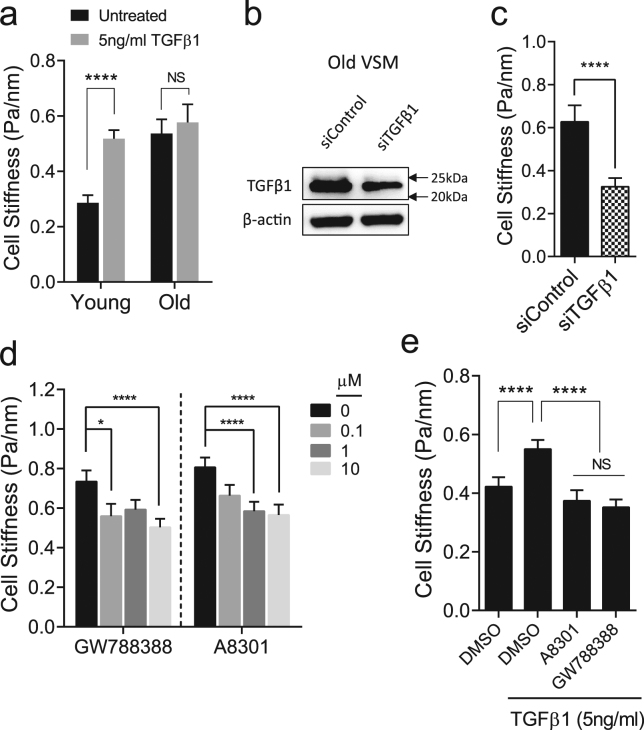
Functional changes in the cytoskeletal stiffness by TGFβ1. **(a)** VSM cells (young and old) were treated for 24 h with or without 5 ng/ml TGFβ1, and changes in cell stiffness were measured by MTC. Data are presented as Geometric Mean ± SE (n = 175–410 individual cell measurements). (**b**) Old VSM cells were transfected with 25 nM small interference RNA (siRNA) against TGFβ1. After 48 h of transfection, TGFβ1 protein level was determined by western blot (Full gel/blot is shown in the Supplementary Figure 6). **(c)** Stiffness of old VSM cells transfected with control or siRNA against TGFβ. Data are presented as Geometric Mean ± SE (n = 112–162 individual cell measurements). **(d)** Stiffness of old VSM cells treated for 24 h with TGFβ1 receptor inhibitors, A8301 and GW788388 (0–10 μM). Data are presented as Geometric Mean ± SE (n = 184–338 individual cell measurements). **(e)** TGFβ1-induced cell stiffness in young VSM cells treated for 24 h with or without the inhibitors (10 μM A8301 and 10 μM GW788388). DMSO (0.1%) was used as control. Data are presented as Geometric Mean ± SE (n = 439–477 individual cell measurements). *P < 0.05; ****P < 0.0001.

At baseline condition, old VSM cells showed appreciable increases in the filamentous actin (F-actin) and the expression of focal adhesion protein vinculin than young VSM cells (Fig. 3). In young VSM cells, exogenous addition of TGFβ1 increased F-actin polymerization (Fig. 3a,b) and stiffened the cytoskeletal network (Fig. 4a), which were comparable to old VSM cells. TGFβ1 did not significantly modify the cytoskeletal structure and function in old VSM cells (Figs 3a,c and 4a). Of note, old VSM cells treated with TGFβ1 exhibited markedly attenuated and delayed reduction in cytoskeletal stiffness in response to cytochalasin-D, an inhibitor of F-actin polymerization (Suppl. Fig. 4a). On the same time scale, blebbistatin, a myosin ATPase inhibitor, did not significantly modify the stiffness of young *vs*. old VSM cells, even in the presence of TGFβ1 (Suppl. Fig. 4a). These results suggest that TGFβ1 reinforces the mechanical phenotype of arterial aging in VSM cells through a long-range regulation of the actin CSK network.

Consistent with this structure-function relationship, siRNA-mediated knockdown of TGFβ1 decreased the stiffness of old VSM cells (Fig. 4b,c). In addition, pharmacological inhibition of the canonical TGFβ1 receptor-Smad2/3 signaling with GW788388 and A-83–01 decreased the stiffness of old VSM cells in a concentration-dependent manner (Fig. 4d). In young VSM cells, GW788388 and A-83-01 effectively inhibited, in turn, TGFβ1-induced Smad2/3 phosphorylation (Suppl. Fig. 5) and TGFβ1-induced cell stiffening (Fig. 4e). Collectively, these results suggest that arterial aging modifies the stiffness of VSM cells and that this mechanical transgression deep within the cell body (i.e. cytoskeletal structures) is regulated by TGFβ1 via its canonical signal transduction pathways.

### TGFβ1 modifies the expression of mechanosensitive integrin receptors

The cell surface integrin receptors play a critical role in sensing the external molecular microenvironment, as well as transducing these mechanical and/or chemical cues into physical forces that are necessary for cell adhesion, migration, contraction, and proliferation. Using RT-PCR, we assessed the transcript levels of α and β integrin subunits that when dimerized bind to ECM proteins of the vascular wall (i.e. collagen, laminin, and fibronectin)^8,12,13^, as well as α_v_β_6_ which is reported to physically liberate TGFβ1 from its latency^37–40^. Although old VSM cells showed appreciable increases in the mRNA levels of α_3_, α_7_, β_3_ and β_5_ integrin subunits than young VSM cells, we did not detect noticeable differences in the transcript levels of integrin subunits responsible for binding to collagen (α_1_β_1_, α_2_β_1_) and fibronectin (α_5_β_1,_ α_v_β_3_), as well as for activating the pro-TGFβ1 (α_v_β_6_) (Suppl. Fig. 7, untreated cells).

Interestingly, in both young and old VSM cells, exogenous addition of TGFβ1 decreased the mRNA levels of integrin α subunits (α_1_, α_2_, α_3_, and α_7_) responsible for binding to collagen and/or laminin whereas increased α_5_ and α_v_ integrin subunits for binding to fibronectin (Suppl. Fig. 7). In young VSM cells, TGFβ1 treatment also increased the mRNA levels of β_1_ and β_6_ integrin subunits (Suppl. Fig. 7). Due to the poor sensitivity of the commercially available antibodies, we could only confirm the protein abundance of α_5_β_1_, but not α_v_β_3_ or α_v_β_6_, by western blot (Suppl. Fig. 8a). As shown in Supplementary Figure 8b, though, isolated primary VSM cells stained positive for α_5_β_1_ and α_v_β_3_. At baseline, the fluorescence intensities of α_5_β_1_ and α_v_β_3_ were appreciably higher in old VSM cells than young VSM cells. By contrast, young VSM cells treated with TGFβ1 showed comparable α_5_β_1_ and α_v_β_3_ staining to that of old VSM cells (Suppl. Fig. 8b). Taken together, these series of studies showed that TGFβ1 expression, or TGFβ1 receptor activation, in VSM cells increases the expression of α_5_β_1_ and α_v_β_3_–the primary integrin receptors for fibronectin^41,42^.

### TGFβ1 reinforces the molecular tension in VSM cells through a cluster of α_5_β_1_ and α_v_β_3_ integrins

In order to assess the extent to which TGFβ1-induced VSM stiffening is reinforced through these mechanosensitive fibronectin-specific integrin receptors, we next applied single-molecule ‘tension gauge tether (TGT)’ method (Suppl. Fig. 9)^43,44^. TGT is a rupturable double stranded DNA (dsDNA), which is designed to rupture under a critical force (T_tol_, tension tolerance). T_tol_ is controlled by biotins precisely located on the bottom DNA strand; and, the molecular tension of a single receptor-ligand bond is characterized through a specific receptor motifs conjugated to the upper DNA strand (Suppl. Fig. 9). For these studies, we used cyclic-RGDfK pentapeptide, which is the receptor motif for α_5_β_1_ and α_v_β_3_^43,44^.

At short times (30 min), isolated VSM cells from both adult and aged rats adhered and spread on the 54 pN TGT surface, but did not pull apart dsDNA tethers (Suppl. Fig. 10; no loss of fluorescence signal in the Cy3 channel). These results are consistent with previous studies showing a universal molecular force of a single integrin receptor being ~40 pN tension^44^. As such, an individual cell is able to form a stable adhesion on 54 pN TGT because T_tol_ = 54 pN is sufficient to bear the pulling force of a single ligand-integrin bond exerted by a living cell. In contrast, the extent of cellular adhesion and spreading was short-lived and remarkably low on the 23 pN TGT surface (Suppl. Fig. 10a,b).

Of note, whereas old VSM cells were less adhered to 23 pN TGT than young VSM cells (Suppl. Fig. 10a), they were able to pull apart dsDNA tethers from the surface, marking behind clearly visible ‘entire’ rupture patterns (i.e. loss of fluorescence signal at the ligand-receptor contact area)^44^ (Suppl. Fig. 10c). In contrast, young VSM cells adhered to the 23 pN TGT surface produced ruptures that were smaller in magnitude and predominantly localized at the cell periphery (edge’ ruptures)^44^ (Suppl. Fig. 10c). Interestingly, young VSM cells treated with TGFβ1 became less adhered to the 23 pN TGT surface and, as did old VSM cells, made ‘entire’ rupture patterns (Suppl. Fig. 10a–c).

**Figure 5:**
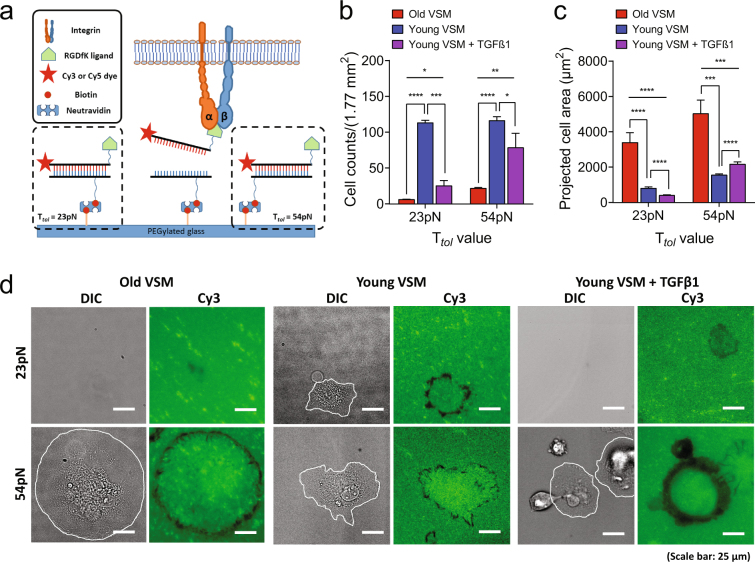
Cell adhesion and spreading on TGT surfaces (incubation time, t = 2 h). **(a)** A schematic of TGT assay. (**b**) Total number of adherent VSM cells on 23 and 54 pN TGT surfaces. The number of adherent cells after 2 h incubation was counted. The TGT assay in each condition was repeated at least three times. Data are presented as Mean ± SE (n = 3 or 4). (**c**) Measured projected area of each adherent cell on 23 and 54 pN TGT surfaces. Data are presented as Mean ± SE. On 23 pN TGT surface: n = 19, old VSM; n = 37, young VSM; n = 61, young VSM with TGFβ1. On 54 pN TGT surface: n = 25, old VSM; n = 171, young VSM; n = 348, young VSM with TGFβ1. (**d**) TGT rupture patterns marked by an individual VSM cell. Cell boundary is drawn in white line. dsDNA tethers rupture (fluorescence signal loss in the Cy3 channel) when a stronger molecular tension above a tension tolerance (T_tol_) is applied on a receptor-ligand bond. Scale bar is 25 µm. *P < 0.05; **P < 0.01; ***P < 0.001; ****P < 0.0001.

At longer time (t = 2 h), isolated VSM cells derived from both adult and aged rats adhered and spread on the 23–54 pN TGT surface (Fig. 5a–d). Although old VSM cells were less adhered on the surface (Fig. 5b, on both 23 pN and 54 pN TGT), the adherent cells were significantly bigger than young VSM cells (Fig. 5c). Of note, old VSM cells produced ‘entire’ ruptures on 23 pN TGT, as seen at short time (Suppl. Fig. 10c), while young VSM cells made ruptures that were predominantly localized to the cell periphery (‘edge’ ruptures)^44^ (Fig. 5d). On the other hand, both young and old VSM cells marked ‘edge’ ruptures on the 54 pN TGT surface; however, the ruptures were more pronounced in old VSM cells (Fig. 5d; i.e. bigger rupture widths). Strikingly, similar to old VSM cells, young VSM cells treated with TGFβ1 marked bigger dsDNA ruptures at the cell periphery (Fig. 5) that corresponded, in turn, to the site of focal adhesions (Suppl. Fig. 11).

Taken together, these results revealed a discordant spatiotemporal evolution of molecular tension in young *vs*. old VSM cells and that this short-range physical force at the single receptor-ligand bond is reinforced by TGFβ1. The data also showed that, within the early stages of cell adhesion, the single-molecule force exerted by an individual young VSM cell through α_5_β_1_ and/or α_v_β_3_ is less than 23 pN, and increased upon TGFβ1 treatment, to an equivalent molecular tension of an old VSM cell–i.e. greater than 23 pN, but less than 54 pN.

### Large-scale stress field in aging VSM cells

**Figure 6:**
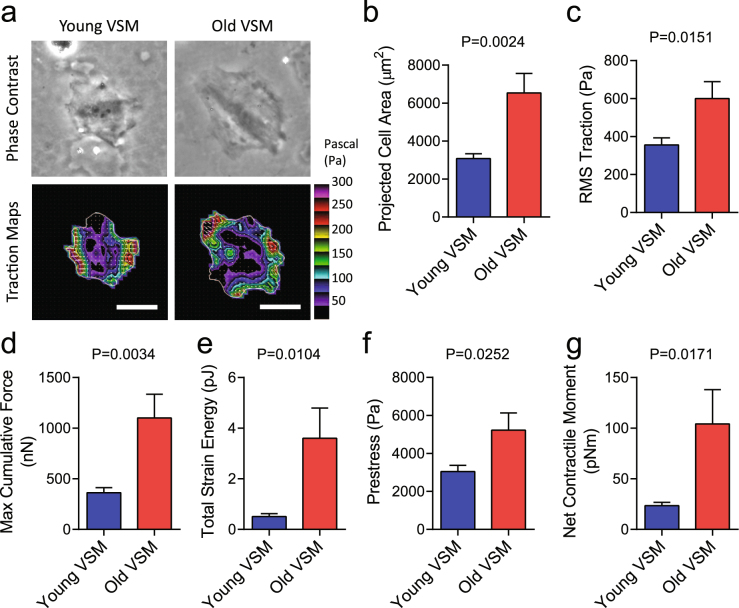
Traction stress maps of isolated VSM cells. **(a)** Representative phase contrast and traction field images of young *vs*. old VSM cells adhered to an elastic gel coated with type I collagen (Young’s modulus of 8 kPa with a Poisson’s ratio of 0.48). The white lines show the cell boundary, colors show the magnitude of the tractions in Pascal (Pa) indexed to the color bar at the right, and arrows show the direction and relative magnitude of the tractions. Scale bar is 50 μm. As described^45,48^, for each individual adherent cell, we computed **(b)** projected area; **(c)** root mean square (RMS) traction; (**d**) maximum cumulative force; **(e)** total strain energy; (**f**) prestress; and, (**g**) net contractile moments. Data are presented as Mean ± SE (n = 8–10 individual cells per group).

To assess the extent to which single-molecule integrin pulling force is transmitted to large-scale cellular behaviors, we measured the traction stress arising at the interface between each adherent cell and the elastic matrix using Fourier transform traction microscopy (Fig. 6, Young’s modulus of ~8 kPa). Compared with young VSM cells, old VSM cells were appreciably bigger in size (Fig. 6a,b) and showed marked increases in traction (root mean square) average over the entire cell projected area (Fig. 6c). The average traction stress generation of old VSM cells (599.5 ± 89.1 Pa; Mean ± SE, *n* = 8) was ~68% greater than that of young VSM cells (356.0 ± 37.2 Pa; Mean ± SE, *n = *10). From the computed traction stress, we also derived a number of other metrics of intracellular force, including the maximum cumulative force, strain energy imparted by the cell to the substrate, the tensional stress borne by actin microfilaments, and the amplitude of net contractile moment (Fig. 6d–g). All computed physical metrics of forces were significantly greater in old VSM cells than young VSM cells. In particular, compared with young VSM cells, old VSM cells showed an approximately four-fold higher net contractile moment (104.1 ± 33.9 *vs*. 23.5 ± 3.2 pNm; P = 0.0171), which is a scalar measure of the cell’s contractile strength^45^.

Of note, both basal tone and TGFβ1-induced stress were appreciably higher in old VSM cells than young VSM cells and irrespective of the matrix stiffness (Suppl. Fig. 12, Young’s Modulus of ~20 kPa). Taken together, these data established that molecular tension of greater than 54 pN is concentrated at an individual integrin-focal adhesion cluster and is propagated, at long distance, through a tense network of internal cytoskeleton to generate stress field in isolated primary VSM cells. Importantly, these results demonstrated that such long-range regulation of cytoskeletal structures is modified by arterial aging and is solidified by TGFβ1 through a cluster of mechanosensitive α_5_β_1_ and α_v_β_3_ integrins.

## Conclusion

With each beat of the heart, and each thrust of the stroke volume, an elastic response of the central arteries fashions, in turn, cardiovascular health and long-lived human physiology. With aging, however, there is a general breakdown of vascular distensibility/elasticity which is marked by inflammation, fibrosis and stiffening in the arterial wall. Using an experimental model of arterial aging, here we identified age-associated stiffening of the VSM. This phenotypic expression of increased cytoskeletal stiffness in old VSM cells was maintained in culture and persistent across a wide range of matrix rigidity. Using siRNA-mediated knockdown of TGFβ1, and pharmacological inhibition of TGFβ1 receptor signal transduction, we further demonstrated that this long-range material stiffness of the actin cytoskeletal network is positively associated with TGFβ1 expression and activation and, on multiple time and length scales, reinforced through a cluster of mechanosensitive integrin receptors α_5_β_1_ and α_v_β_3_. Taken together, these studies serve as a proof-of-concept that the broad-based inhibition of TGFβ1 expression, or activation, targeted at the structural cell-types of VSM may be a useful therapeutic approach to mitigate the pathologic progression of central arterial wall stiffening associated with aging.

## Materials and Methods

### Material and reagents

Human recombinant TGFβ1 was purchased from R&D systems (Catalog # 204B), and TGFβ1 type I receptor inhibitors, A-83-01 and GW788388, were purchased from Sigma-Aldrich (Catalog # SML0788 and Catalog # SML0116). All primary antibodies and HRP-conjugated goat anti-rabbit IgG used in this study were obtained from Cell Signaling Technology and used as directed by the manufacturer’s recommendations.

### Animals

Male Fisher 344 crossbred Brown Norway rats (F344XBN; 8-month-old and 30-month-old) were obtained from the National Institute on Aging Contract Colonies (Harlan Sprague Dawley, Inc., Indianapolis, IN). The animal protocol used was approved by the Institutional Animal Care and Use Committee of the Gerontology Research Center and complied with the guide for the care and use of laboratory animals (NIH publication No. 3040-2, revised 1999).

### VSM cell isolation and culture

Vascular smooth muscle (VSM) cells were enzymatically isolated and cultured as previously described^46,47^. Briefly, F344XBN rat thoracic aortas were rinsed in Hanks balanced salt solution (HBSS) containing 50 μg/mL penicillin, 50 μg/mL streptomycin and 0.25 μg/mL amphotericin B (Gibco). After digestion for 30 min with 2 mg/mL collagenase I solution (Worthington Biomedical, Freehold, New Jersey) at 37 °C, the adventitia and intima layers were removed, and the vessel media was further digested with 2 mg/mL collagenase II/0.5 mg/mL elastase (Sigma) for 1 h at 37 °C. The isolated cells were washed and plated in complete medium. In all cases, >95% of cells stained positive for α-smooth muscle actin (α-SMA) and smooth muscle myosin heavy chains (SM1 and SM2) (Suppl. Fig. 1b,c). Cells were maintained with VSMC medium including: DMEM (Gibco), 10% FBS (Sigma), 1% NEAA and 1% penicillin/streptomycin (Gibco). For all studies, we used early passage cells (passages 1–3).

### Live cell micromechanical methods

Using *spontaneous* and *forced* motions of ferrimagnetic microbeads (~4.5 μm in diameter) functionalized to the living CSK, we detected the remodeling dynamics and the material properties of individual primary VSM cells isolated from the thoracic aorta of adult (8 months) *vs*. aged (30 months) F334XBN rats. These methods, Spontaneous Nanoscale Tracer Motions (SNTM) and Magnetic Twisting Cytometry (MTC) are described in detail elsewhere^26,29,31,32,34^. For these studies, VSM cells were plated on collagen-coated plastic tissue culture 96-wells (30,000 cells/well), or, plated on collagen-coated gel blocks of varying rigidity (~1 to 20 kPa; 150,000 cells/block). Unless otherwise noted, prior to cell micromechanical measurements, cells were incubated for 24 h with medium containing 1% FBS: all measurements were made in 1% FBS containing medium.

First, using SNTM, we visualized *spontaneous* nanoscale displacements of an individual functionalized bead (~50–100 beads per field-of-view) and recorded its positions at frequency of 12 frames/s for *t*_max_ ~300 s via a CCD camera (Orca II-ER, Hamamatsu, Japan) attached to an inverted optical microscope (Leica Microsystems, Bannockburn, IL). The trajectories of bead motions in two dimensions were then characterized by computing the mean square displacement of all beads as function of time [MSD(t)] (nm^2^), as previously described^29^. Herein, we analyzed MSD data for times greater than 10 s and up to 300 s. For individual cell-wells and experimental conditions, diffusion coefficient D* and the exponent α of the bead motion were also estimated from a least-square fit of a power-law to the ensemble average of MSD data versus time.

We then applied *forced* motions of the same functionalized beads using MTC^26,34^ and measured stiffness (elastic) and frictional (loss) moduli of adherent VSM cells. In brief, the ferrimagnetic beads bound to the underlying CSK were magnetized horizontally with a brief 1,000-Gauss pulse and twisted in a vertically aligned homogeneous magnetic field (20 Gauss) that was varying sinusoidally in time. This sinusoidal twisting magnetic field caused both a rotation and a pivoting displacement of the bead: as the bead moves, the cell develops internal stresses which in turn resist bead motions^34^. Lateral bead displacements in response to the resulting oscillatory torque were detected with an accuracy of 5 nm using an intensity-weighted center-of-mass algorithm^34^. We defined the ratio of specific applied torque to lateral bead displacements as the complex elastic modulus of the cell, ${g}^{\ast }(f)=g^{\prime} (f)+ig^{\prime\prime} (f),$ where *g’* is the storage modulus (cell stiffness), *g”* is the loss modulus (cell friction), and *i*^2^ = −1^34^. Cell stiffness and friction are expressed in units of Pascal per nm (Pa/nm).

### Fourier Transform Traction Microscope (FTTM)

A detailed description of this technique is provided by Butler and colleagues^45,48^ and in our previous study^26^. In brief, cells were plated sparsely on elastic gel blocks (~1,500 cells per gel block) coated with collagen type I, and allowed to adhere and stabilize for 24 h. For each adherent cell, images of fluorescent microbeads (0.2 μm in diameter, Molecular Probes, Eugene, OR) embedded near the gel apical surface was taken at different times; the fluorescent image of the same region of the gel after cell detachment with trypsin was used as the reference (traction-free) image. The displacement field between a pair of images was then obtained by identifying the coordinates of the peak of the cross-correlation function^45,48^. From the displacement field and known elastic properties of the gel, the traction field was computed using both constrained and unconstrained Fourier transform traction cytometry^45,48^. For these studies, we tuned the elastic gels in a physiological range of arterial wall stiffness (i.e. Young’s moduli of 8 kPa to 20 kPa with Poisson’s ratio of 0.48).

### Tension Gauge Tether (TGT) synthesis

Tension gauge tether (TGT) was fabricated as described^44^. Cyclic peptide RGDfK (cRGDfK, PCI-3696-PI, Peptides International, Inc) was conjugated to the 3′ end of an 18-nucleotide single stranded DNA (ssDNA; 5-/5Cy3/GGC CCG CAG CGA CCA CCC/3ThioMC3-D/3) using a hetero-bifunctional cross-linker (Sulfo-SMCC, Thermo Fisher Scientific Inc.). The Sulfo-SMCC has a maleimide group reacting with a thiol group on the thiol modified ssDNA, as well as a NHS ester group reacting with an amine group on cRGDfK. These functional groups result in the cRGDfK conjugated ssDNA. Next, a complementary ssDNA (5-GGG TGG TCG CTG CGG GCC-3) with biotin at different locations determining different tension tolerances (T_tol_, 23 pN and 54 pN) was annealed to the cRGDfK conjugated ssDNA (as depicted in the Suppl. Fig. 9).

### Cell adhesion on TGT surfaces

To determine molecular tension exerted on a single integrin-ligand bond during VSM cell adhesion, TGT surfaces were prepared following a protocol used in our previous works^44^. 1 µM TGTs with different tension tolerances (23 and 54 pN) were immobilized on a PEGylated glass slide via a neutravidin-biotin interaction. Since the PEGylated glass surface is a non-fouling surface, cell adhesion was allowed only at the TGT coated region. VSM cells were collected from a culture dish and re-dispersed in a serum-free DMEM medium. The cells were loaded onto the TGT surfaces at the density of 3.75 × 10^4^ cells/ml. After 30 min or 2 h incubation at 37 °C, the cells were fixed using a 4% para-formaldehyde solution. DIC images of the fixed cells and fluorescence images of the TGT rupture patterns were taken by an epi-fluorescence microscope (Nikon Ti-E, Nikon Inc.). Projected area and the number of adherent cells on each TGT surface were analyzed using ImageJ.

### Immunostaining of integrin α_5_β_1_ and α_v_β_3_ expressed on VSM cells

Expression levels of α_5_β_1_ and α_v_β_3_ integrins on VSM cells were characterized by immunostaining. In brief, the cells were fixed with a 4% para-formaldehyde solution for 10 min and washed with a PBS solution three times. The cells were permeabilized by a 0.5% Triton-X solution, and incubated with either anti-integrin α_5_β_1_ antibody (MAB1969, Millipore, USA) or anti-integrin α_v_β_3_ antibody (sc-7312 AF488, Santa Cruz Biotechnology, USA). FITC-conjugated secondary antibody (Goat anti-mouse antibody-FITC, F-2761, Thermo Fisher Scientific, Inc.) was then added for the fluorescence imaging. The fluorescence intensity of α_5_β_1_ or α_v_β_3_ integrin in an individual cell was measured by ImageJ, and the relative intensity was plotted using the Origin software (OriginLab Coporation).

### Immunostaining of focal adhesion and actin cytoskeleton

Similar to the integrin staining described above, vinculin was stained by sequential addition of primary and secondary antibodies using a commercial focal adhesion staining kit (FAK100, Millipore). Simultaneously, actin filaments were stained by TRITC-labeled Phalloidin, and the nucleus was stained by DAPI. Focal adhesion, actin, and DAPI in the fixed VSM cells adhered to the TGT surfaces were visualized by an epi-fluorescence microscope (Nikon Ti-E, Nikon Inc.).

### Enzyme-linked immunosorbent assay for TGFβ1

Enzyme-linked immunosorbent assay kit for detection of TGFβ1 was obtained from R&D Systems and experiments followed the manufacturer’s suggested guidelines and as described previously^49^. In brief, young and old VSM cells were cultured in 25-cm^2^ flask and subsequently serum deprived for 12 h. Serum free supernatants from 24-h cultures were collected, and total protein content was determined. Cell culture supernatants were aliquoted and treated or left untreated with 1 N HCl for 10 min to activate latent TGFβ1 to the immunoreactive form. Standard curve was generated using serial dilutions of human recombinant TGFβ1. Data are expressed as a pg of active TGFβ1 per mg of total protein in cell culture supernatants.

### Western Blotting

The western blot was performed using an ECL kit (Thermo Scientific) based on the manufacturer’s recommendations. In brief, samples were boiled in 1 × SDS loading buffer, separated by SDS-PAGE gels, and transferred onto a nitrocellulose membrane, which was blocked with 5% non-fat dry milk or bovine serum albumin (BSA) followed by incubation with primary antibodies at 4 °C overnight. For these studies, we used rabbit anti-phospo-smad2 antibody (1:500), rabbit anti-smad2 antibody (1:1,000), rabbit anti-phospho-smad3 antibody (1:500), rabbit anti-smad3 antibody (1:1,000). HRP-conjugated goat anti-rabbit IgG was used as secondary antibody (1:10,000); and, signals were detected using Pierce ECL Western Blotting Substrate (Thermo Scientific). GAPDH (rabbit anti-GAPDH antibody, 1:4000) was used as a loading control.

### Data analysis

All quantitative data were derived from multiple independent experiments, and statistical differences were determined by Student’s *t* test (comparison of two sample means) or ANOVA (comparison of more than two sample means). In order to satisfy the distributional assumptions associated Student’s *t* test and ANOVA, cell stiffness data were first converted to log scale prior to analyses. For normally distributed data, the comparisons were performed with Student’s *t* test and one-way ANOVA, followed by Bonferroni post hoc testing. The 2-sided P-values less than 0.05 were considered significant.

## Electronic supplementary material

Supplementary Information
