# Supplementary material for: TGFβ1 reinforces arterial aging in the vascular smooth muscle cell through a long-range regulation of the cytoskeletal stiffness
Source: Sci Rep. 2018 Feb 8;8:2668. doi: 10.1038/s41598-018-20763-w (PMC5805716; doi:10.1038/s41598-018-20763-w)
Supplement: Supplementary file 1 — Supplementary Information [file 41598_2018_20763_MOESM1_ESM.pdf]

Supplementary Information

TGFβ1 reinforces arterial aging in the vascular smooth muscle cell through a long-range regulation of the cytoskeletal stiffness

Wanqu Zhu[1,*], Byoung Choul Kim[2,3,4,5,*], Mingyi Wang[6,*], Jessie Huang[1], Abraham Isak[1], Natalia M. Bexiga[7], Robert Monticone[6], Taekjip Ha[2,3,4], Edward G. Lakatta[6,†], and Steven S. An[1,8,9,†]

[1]Department of Environmental Health and Engineering, Johns Hopkins Bloomberg School of Public Health, Baltimore, MD 21205, USA; [2]Department of Biophysics and Biophysical Chemistry, Johns Hopkins University, Baltimore, MD 21205, USA; [3]Department of Biomedical Engineering, Johns Hopkins University, Baltimore, MD 21218, USA; [4]Howard Hughes Medical Institute, Baltimore, Maryland 21218, USA; [5]Division of Nano-Bioengineering, Incheon National University, Republic of Korea; [6]Laboratory of Cardiovascular Science, Intramural Research Program, National Institute on Aging, National Institutes of Health, Baltimore, MD 21224, USA; [7]Immunobiological and Biopharmaceutical Laboratory, Department of Pharmaceutical Biochemistry Technology, University of Sao Paulo, Sao Paulo, Brazil; [8]Department of Chemical and Biomolecular Engineering, Johns Hopkins University, Baltimore, MD 21218, USA; [9]Department of Biomedical Engineering, Ulsan National Institute of Science and Technology, Republic of Korea.

*These authors contributed equally to this work.

†Corresponding authors. E-mails: san@jhu.edu (S.S.A.); LakattaE@grc.nia.nih.gov (E.G.L.)

The authors declare no conflict of interest

Author Contributions: S.S.A., M.W., and E.G.L. developed the study concept and design. R.M. established and maintained primary VSM cell cultures. W.Z., A.I., J.H., and N.M.B. performed single-cell mechanics and molecular studies. B.C.K. performed single-molecule biophysics studies. W.Z., J.H., and B.C.K. performed data analysis and interpretation. M.W., T.H., and E.G.L. performed data analysis and interpretation, and manuscript preparation. W.Z., B.C.K., M.W., and S.S.A. wrote the manuscript. S.S.A. and E.G.L. directed all studies.

Acknowledgments: This work was supported by funds from the US National Heart, Lung, and Blood Institute (HL107361 to S.S.A.), the Howard Hughes Medical Institute (HHMI) (B.C.K and T.H), and the National Science Foundation Physics Frontier Center (PHY 1430124 to T.H.). S.S.A was also supported by Discovery Award and Catalyst Award from the Johns Hopkins University.

Figure Legends:

Supplementary Figure 1: Cellular models of physiologic arterial aging in VSM. (**a**) Photomicrographs (X400) of young and old rat aortic wall: elastin fiber (Verhoeff's elastic fiber stain, dark blue) and vascular smooth muscle cells (immunostaining against α-smooth muscle actin, brown color). (**b-c**) Representative immunofluorescence of smooth muscle cell markers (**b**, SM-MHC; **c**, αSMA) in young and old VSM cells. F-actin is stained with phalloidin and nucleus with DAPI. (**d**) Proliferation (measured by cell count) of young and old VSM cells. Data are presented as Mean $\pm$ SD (n = 3).

Supplementary Figure 2: Dynamics of CSK remodeling in isolated VSM cells. (a) Representative bright field image of isolated VSM cells in culture, showing functionalized RGD-coated ferrimagnetic beads (~4.5 μm in diameter). (**b**) The trajectories of *spontaneous* bead motions recorded over the course of 5 min. For clarity, only few representative tracings are depicted from the same point of origin (10X objective, n = 20 beads). (**c**) Computing mean square displacement (MSD) of all beads as function of time *t*. Data are presented as Mean $\pm$ SE (n = 501-563 individual cell measurements). (**d**) Corresponding MSD measured at 10s and 300s, respectively.

Supplementary Figure 3: CSK remodeling and stiffness of isolated VSM cells adhered to elastic substrate with varying rigidity. (**a, c**) Computed MSD of young *vs.* old VSM cells plated on elastic gels (**a**, 1 kPa 'softer'; **c**, 8 kPa 'stiffer') as measured by SNTM. Data are presented as Mean $\pm$ SE (n = 9-15 individual cell-wells, comprising ~878-1346 individual beads measurements). (**b, d**) The elastic modulus g' (stiffness) and the loss modulus g" (friction)

measured over 5 decades of frequency with MTC in adherent VSM cells on (**b)** 'softer' and (**c)** 'stiffer' matrices. Data are presented as Geometric Mean $\pm$ SE (n = 67-370 individual cell measurements).

Supplementary Figure 4: Effects of cytochalasin-D and blebbistatin on the stiffness of isolated VSM cells. VSM cells (young and old) were treated for 24 h with or without 5ng/ml TGFβ1, and dynamic changes in cell stiffness in response to (**a**) cytochalasin-D (1μM, F-actin inhibitor) and (**b**) blebbistatin (10μM, myosin II inhibitor) were measured by MTC. For each individual cell, baseline stiffness was measured for the first 60 s, and after drug addition stiffness was measured continuously for the next 540 s. For each cell, stiffness was normalized to its baseline stiffness before the agonist stimulation. Data are presented as Mean $\pm$ SE (n = 212-321 individual cell measurements per group). **, $P<0.01$; ****, $P<0.0001$.

Supplementary Figure 5: This figure displays the full-length gel/blot for Figure 2b shown in the text/Results and Discussion. The identification of **(a)** Smad2/3 and **(b)** p-Smad2/3 bands was based on the expected molecular weight, which was reported in other publications. Panels **a** and **b** are from the same gel/blot. Membrane was cut before probing for Smad2/3 and GAPDH, and re-blotted using p-Smad2/3 antibodies. The blots also show inhibition of TGFβ1-induced Smad2/3 phosphorylation in cells (young and old VSM) treated with TGFβ1 receptor I inhibitors (10μM A8301 or 10μM GW788388).

Supplementary Figure 6: This figure displays the partial-length cut **(a)** and the full-length **(b)** gels/blots for Figure 4b shown in the text/Results and Discussion using anti-TGFβ1 from Santa

Cruz (SC-146) and R&D Systems (MAB240), respectively. The identification of TGFβ1 bands were based on the expected molecular weight, which were shown in other publications.

Supplementary Figure 7: Integrin profiling in isolated VSM cells. mRNA levels of integrins are expressed relative to young VSM cells. Data are normalized to β-actin and presented as Mean $\pm$ SD (n = 3). #, $P<0.05$ (untreated *vs.* TGFβ1 treated for the respective group); *, $P<0.05$ (young *vs.* old).

Supplementary Figure 8: Expression of $\alpha_5\beta_1$ and $\alpha_v\beta_3$ in isolated VSM cells. **(a)** This figure displays the full-length gel/blot showing protein expression levels of $\alpha_5\beta_1$. The identification of α_5 and β_1 subunit bands was based on the expected molecular weight, which was shown in other publications. The same gel/blot was re-blotted using anti-GAPDH antibody for loading control. **(b)** Immuno-staining of integrin $\alpha_5\beta_1$ and $\alpha_v\beta_3$ expressed in VSM cells. Young VSM cells (with/without 5ng/ml of exogenous TGFβ1) and old VSM cells were stained with an antibody targeting $\alpha_5\beta_1$ or $\alpha_v\beta_3$. Scale bar is 50 µm. Fluorescence intensities of $\alpha_5\beta_1$ and $\alpha_v\beta_3$ are shown in the bar graph below. ****, $P<0.0001$.

Supplementary Figure 9: The principle of TGT assay. **(a)** A schematic of TGT assay. **(b)** Geometries and estimated tolerances of dsDNA tethers (not drawn in scale).

Supplementary Figure 10: Initial cell adhesion on TGT surfaces (incubation time, t = 30 min). **(a)** Total number of adherent VSM cells on 23 and 54 pN TGT surfaces. The number of adherent cells after 30 min incubation was counted. The TGT assay in each condition was

repeated four times. Data are presented as Mean ± SE (n = 4). **(b)** Measured projected area of each adherent VSM cell on 23 and 54 pN TGT surfaces (n = 16-92 individual cells). Data are presented as Mean ± SE. *, $P<0.05$; **, $P<0.01$; ***, $P<0.001$; ****, $P<0.0001$. **(c)** TGT rupture patterns marked by an individual VSM cell at the ligand-receptor contact area. The dashed lines in each DIC image indicate the periphery of each cell. Scale bar is 25 μm.

Supplementary Figure 11: Focal adhesion formation on the 54 pN TGT surface. Actin and vinculin were stained in young VSM cells treated for 24 h with or without 5ng/ml TGFβ1. Focal adhesion was produced predominantly at the cell periphery, and corresponded to TGT ruptures shown in the Cy3 channel. TGFβ1 accelerated focal adhesion formation in the young VSM cells, resulting in higher forces above 54 pN at the location of the focal adhesion.

Supplementary Figure 12: TGFβ1-induced stress maps in isolated VSM cells. VSM cells (young and old) were treated for 24 h with or without 5ng/ml TGFβ1, and traction maps were measured with FTTM. For these studies, we tuned the elastic gels to ~20 kPa to mimic physiological stiffness of arterial wall. Computed root mean square (RMS) traction data are presented as Mean ± SE (n=21-27 individual cells per group). *, $P<0.05$; ***, $P<0.001$.

Suppl. Fig. 1

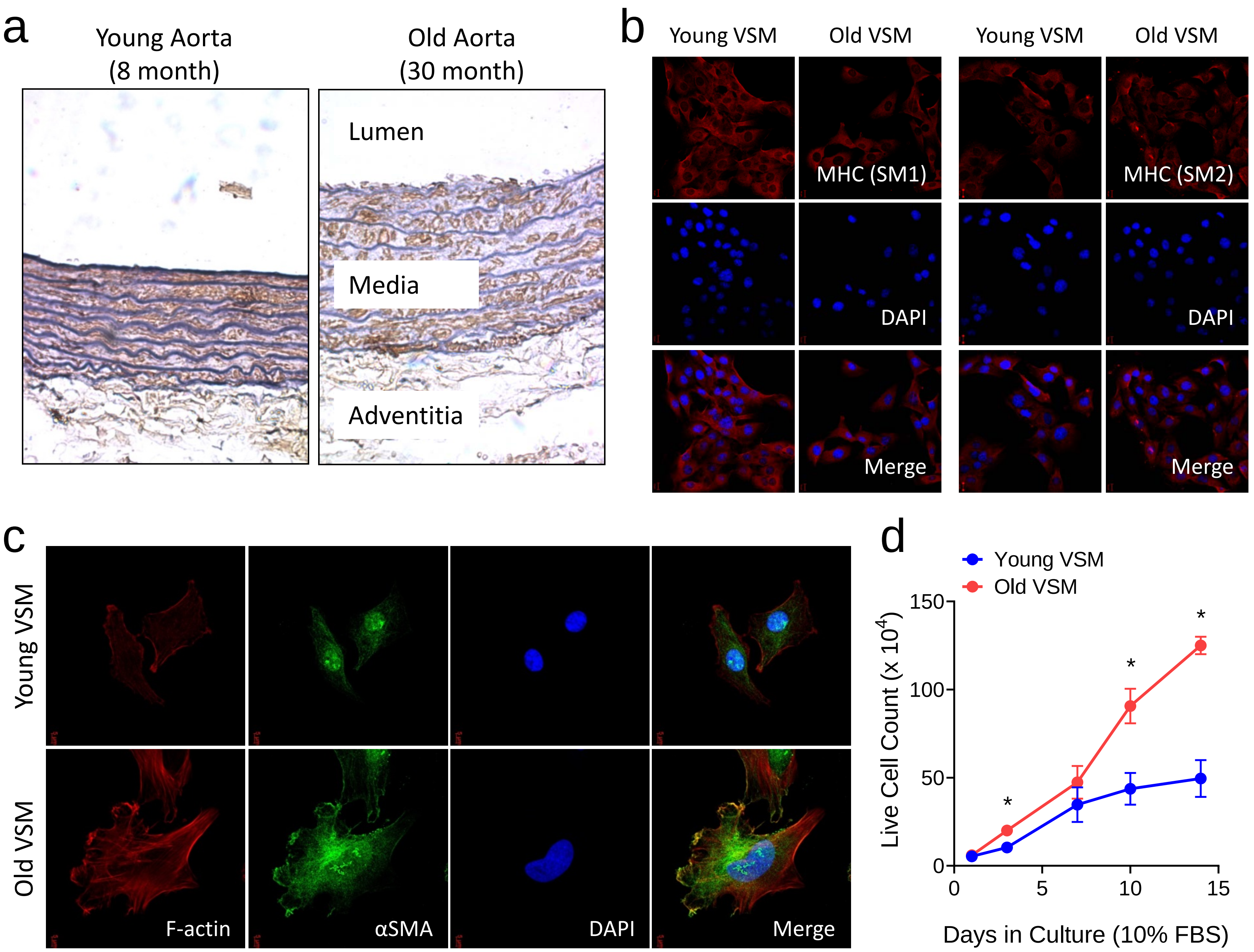
a
Young Aorta
(8 month)
Old Aorta
(30 month)
Lumen
Media
Adventitia
b
Young VSM
Old VSM
Young VSM
Old VSM
MHC (SM1)
MHC (SM2)
DAPI
DAPI
Merge
Merge
c
Young VSM
Old VSM
F-actin
αSMA
DAPI
Merge
d
Young VSM
Old VSM
Live Cell Count (x 10^4)
150
100
50
0
*
*
*
0
5
10
15
Days in Culture (10% FBS)

Suppl. Fig. 2

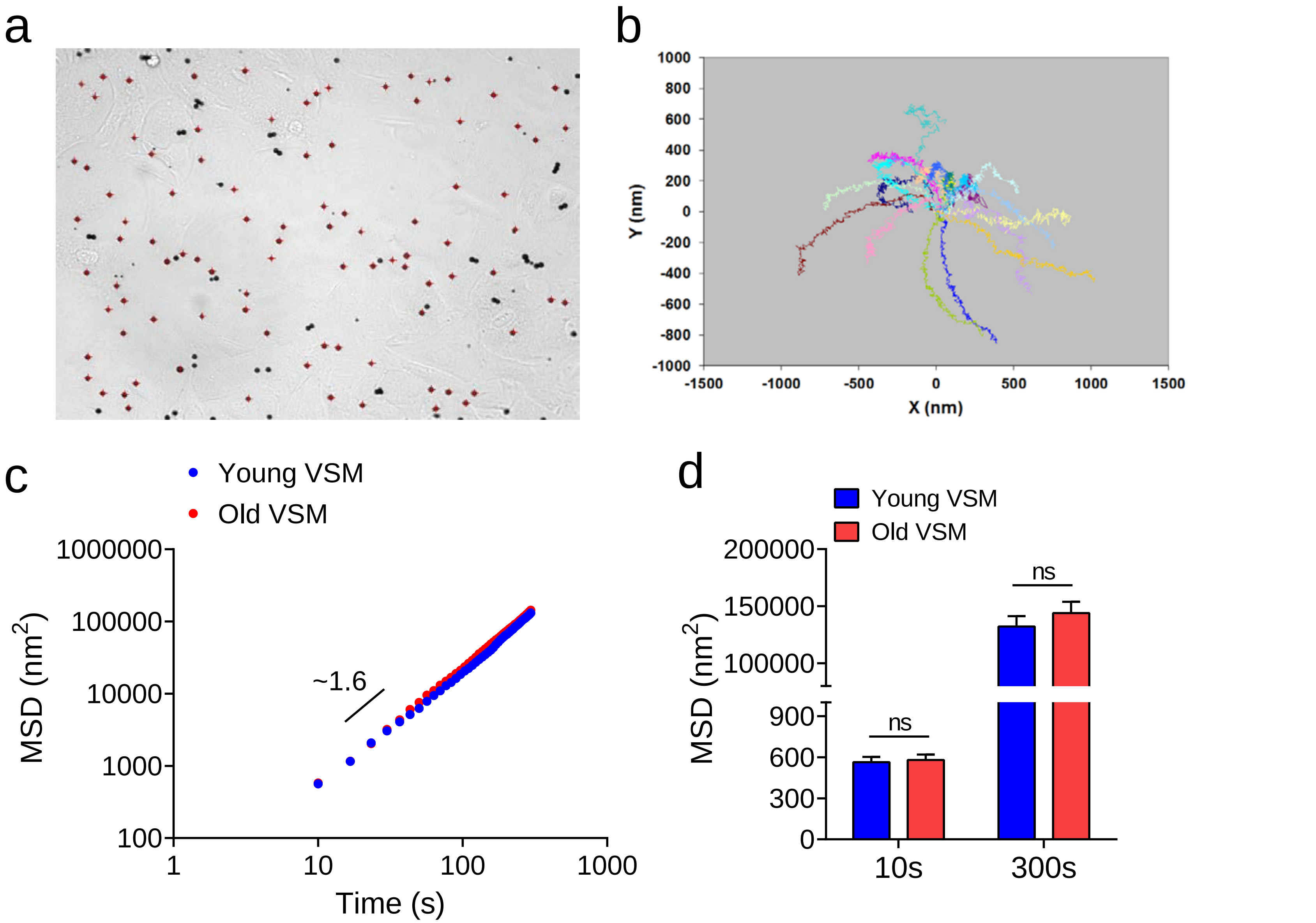
a
b
Y (nm)
X (nm)
c
Young VSM
Old VSM
MSD (nm^2)
Time (s)
~1.6
d
Young VSM
Old VSM
MSD (nm^2)
ns
ns
10s
300s

Suppl. Fig. 3

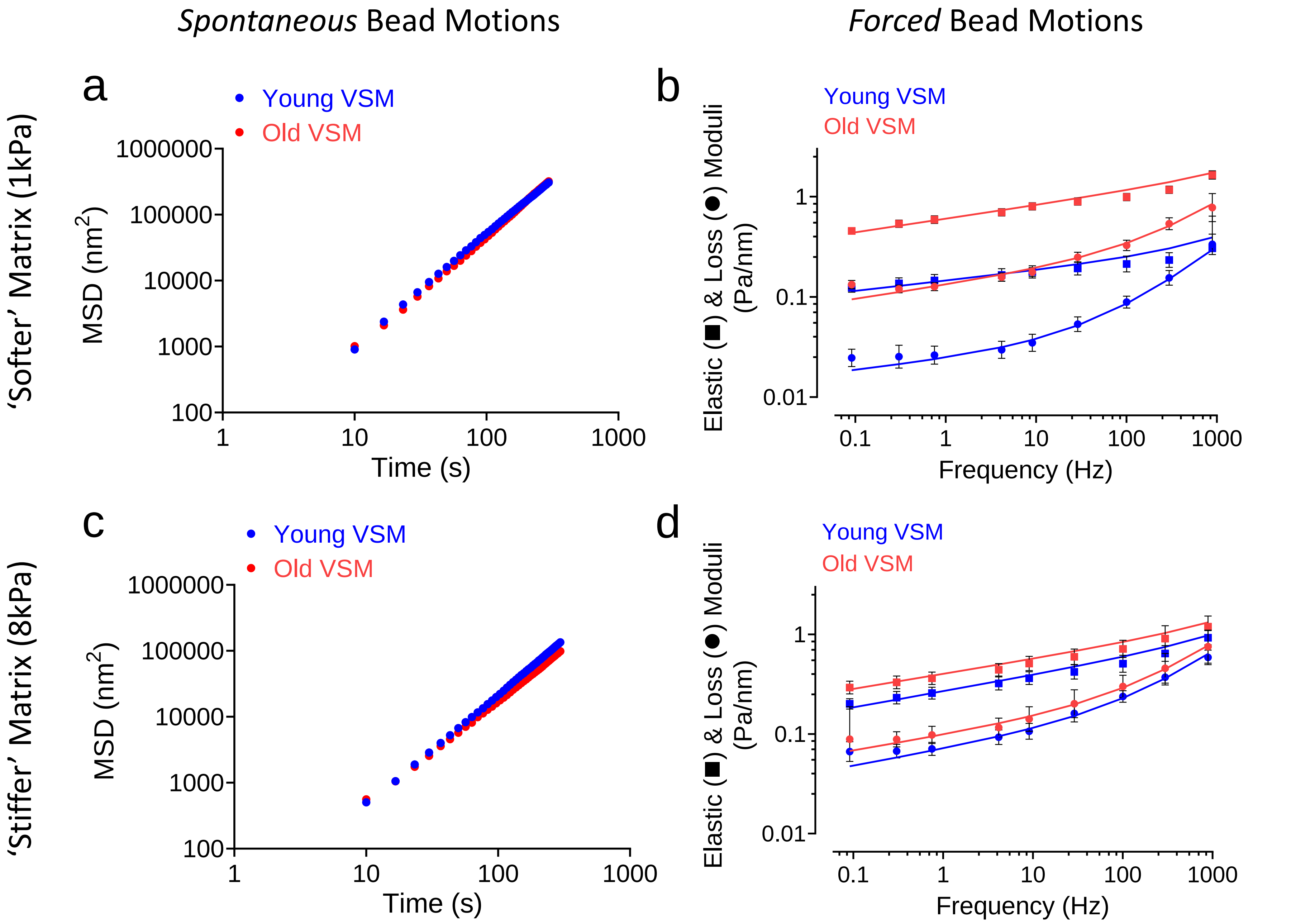

Spontaneous Bead Motions
Forced Bead Motions
a
b
c
d
'Softer' Matrix (1kPa)
'Stiffer' Matrix (8kPa)
Young VSM
Old VSM
MSD (nm2)
Time (s)
1000000
100000
10000
1000
100
1
10
100
1000
Elastic (■) & Loss (●) Moduli (Pa/nm)
Frequency (Hz)
1
0.1
0.01

Suppl. Fig. 4

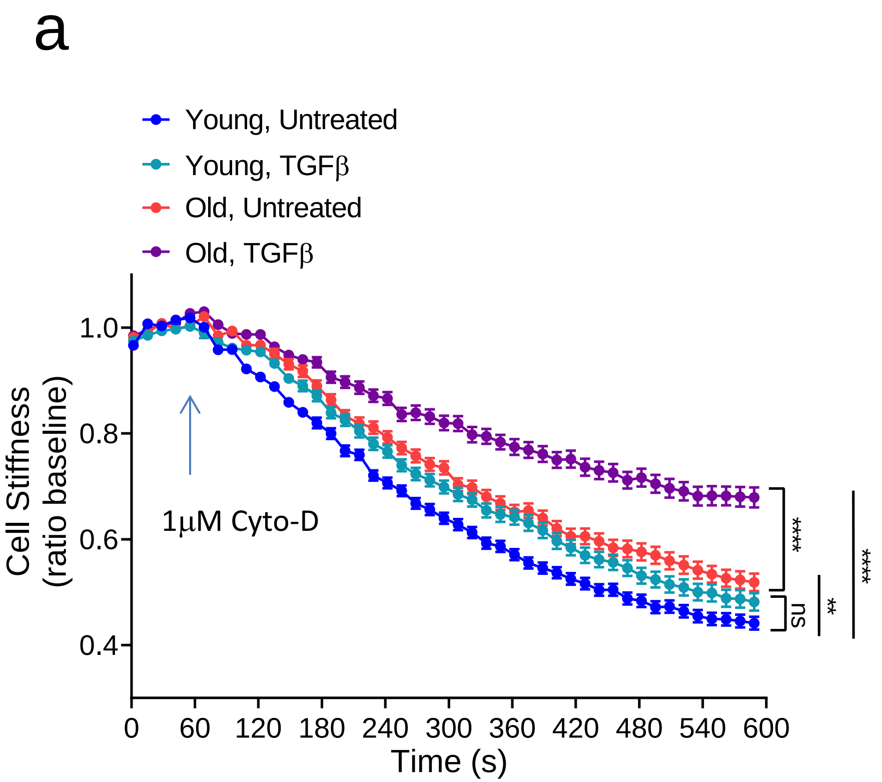
a
Young, Untreated
Young, TGFβ
Old, Untreated
Old, TGFβ
1μM Cyto-D
Cell Stiffness
(ratio baseline)
Time (s)
****
ns
**
****

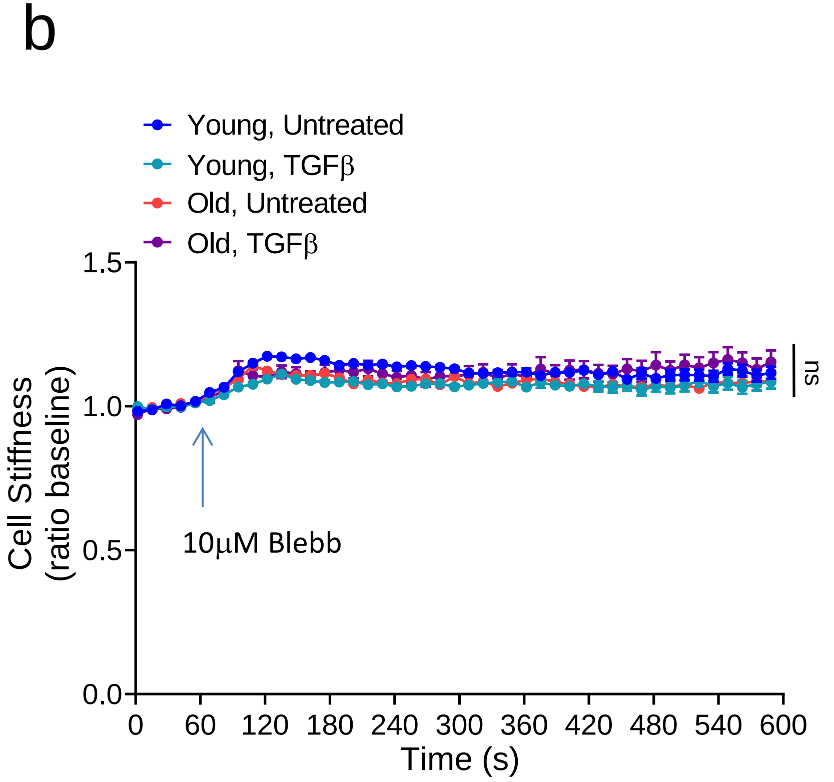
b
Young, Untreated
Young, TGFβ
Old, Untreated
Old, TGFβ
10μM Blebb
Cell Stiffness
(ratio baseline)
Time (s)
ns

Suppl. Fig. 5

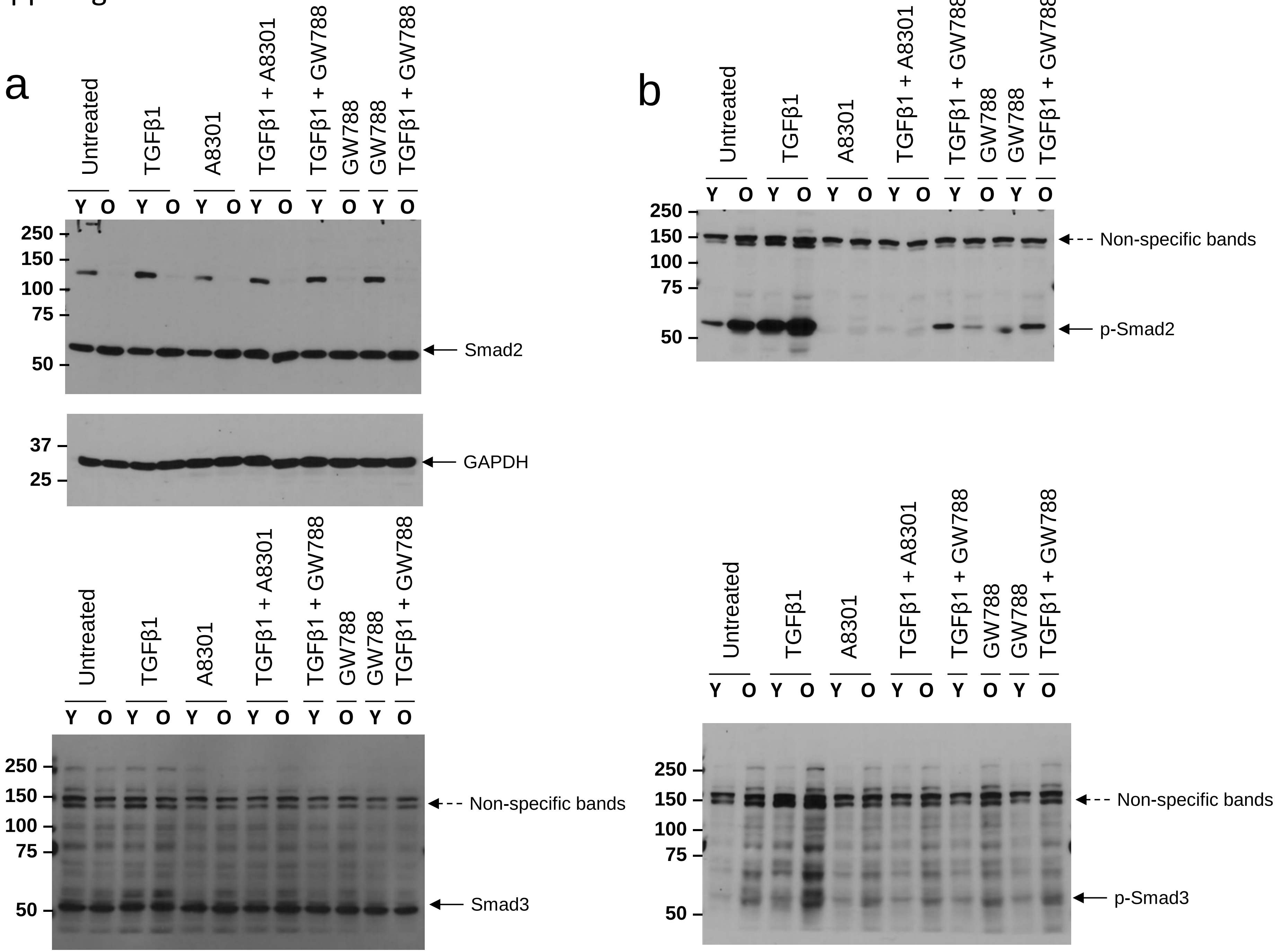
a
Untreated
TGFβ1
A8301
TGFβ1 + A8301
TGFβ1 + GW788
GW788
GW788
TGFβ1 + GW788
Y O Y O Y O Y O Y O Y O
250
150
100
75
50
Smad2
37
25
GAPDH
Non-specific bands
Smad3
b
p-Smad2
p-Smad3

Suppl. Fig. 6

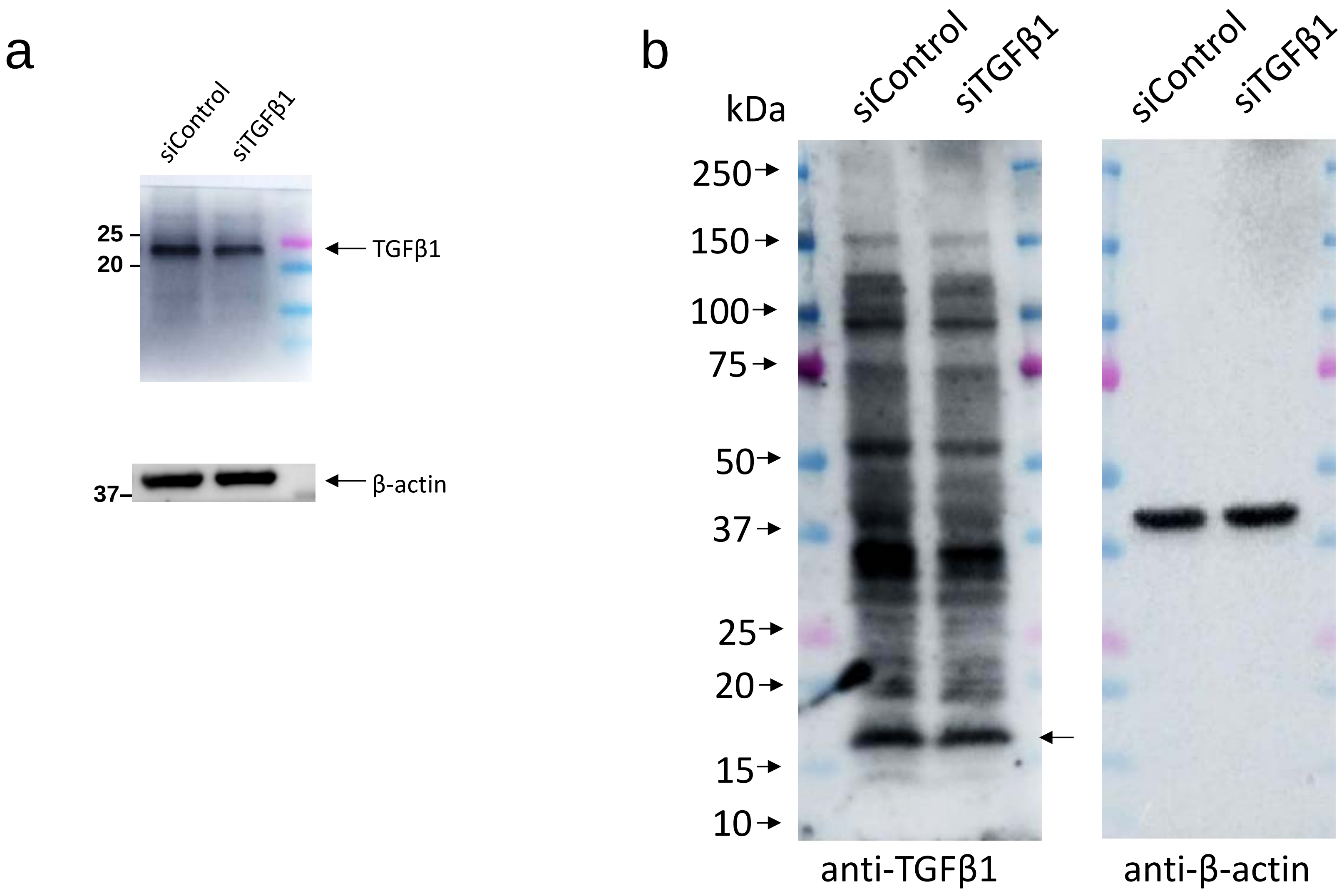
a
siControl
siTGFβ1
25
20
TGFβ1
37
β-actin
b
kDa
siControl
siTGFβ1
siControl
siTGFβ1
250
150
100
75
50
37
25
20
15
10
anti-TGFβ1
anti-β-actin

Suppl. Fig. 7

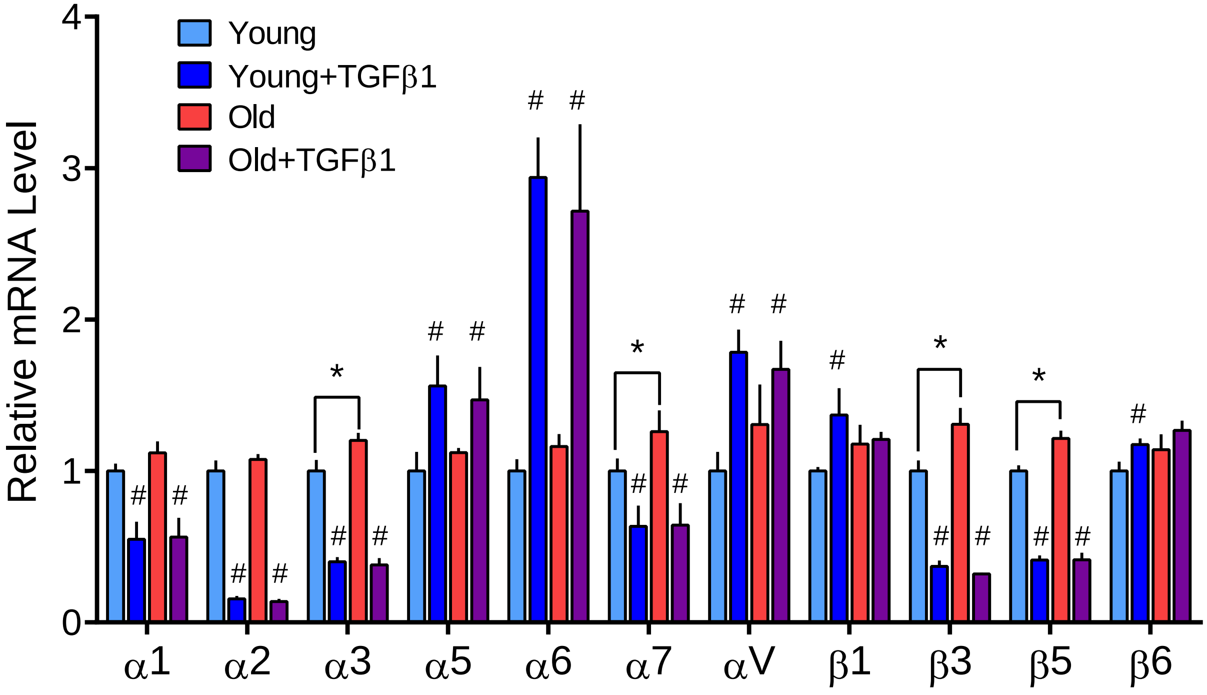

Relative mRNA Level
0
1
2
3
4
Young
Young+TGFβ1
Old
Old+TGFβ1
α1
α2
α3
α5
α6
α7
αV
β1
β3
β5
β6

Suppl. Fig. 8

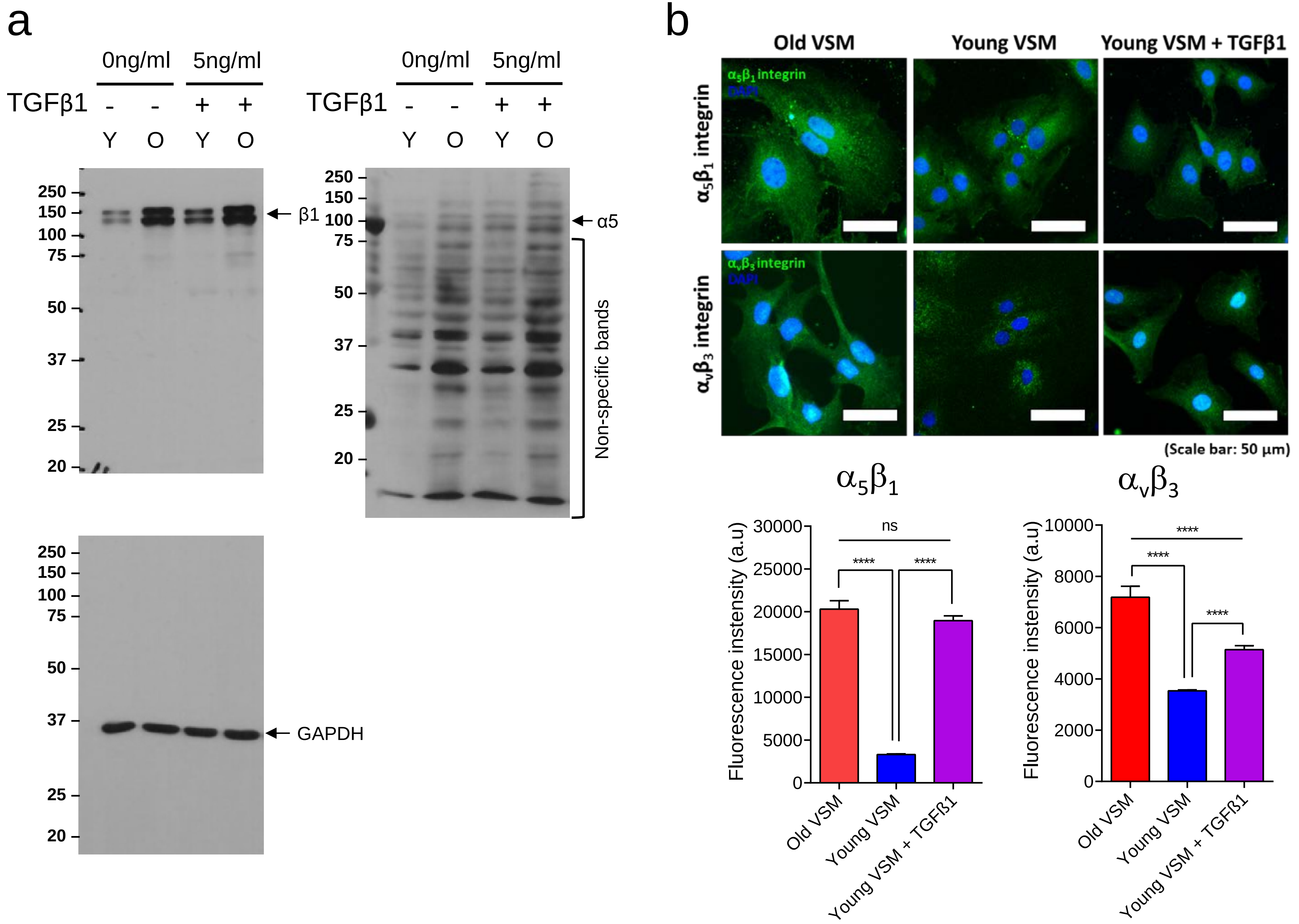
a
0ng/ml
5ng/ml
TGFβ1 - - + +
Y O Y O
250
150
100
75
50
37
25
20
β1
α5
Non-specific bands
GAPDH
b
Old VSM
Young VSM
Young VSM + TGFβ1
α5β1 integrin
αvβ3 integrin
DAPI
(Scale bar: 50 μm)
α5β1
αvβ3
Fluorescence instensity (a.u)
30000
25000
20000
15000
10000
5000
0
10000
8000
6000
4000
2000
ns
****
Old VSM
Young VSM
Young VSM + TGFβ1

Suppl. Fig. 9

Principles of TGT assay: geometries and estimated tension tolerances of TGT

a

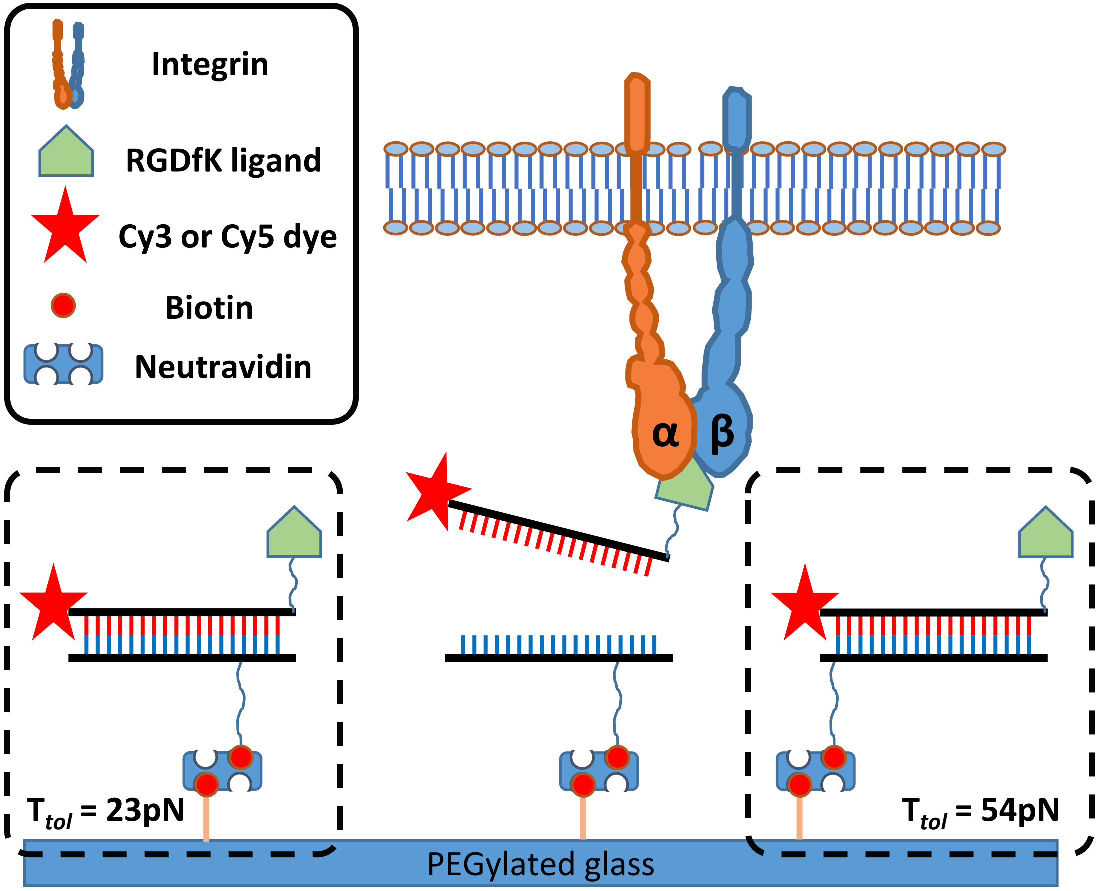

b

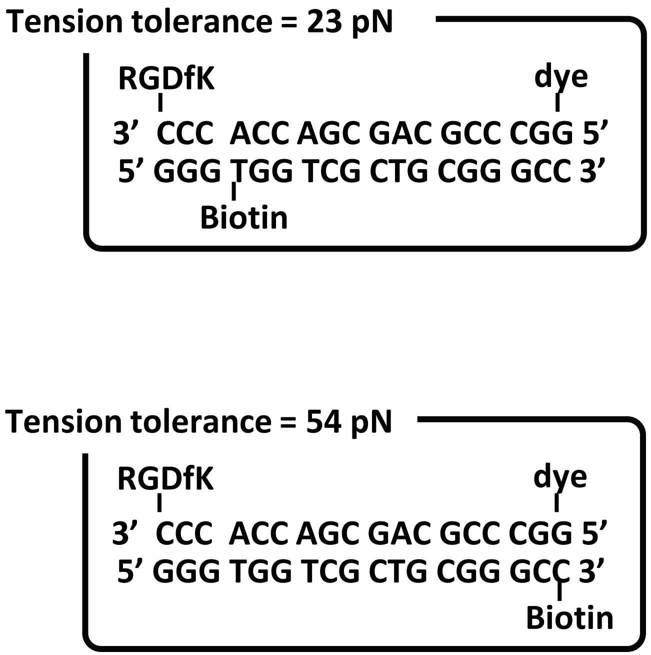

Suppl. Fig. 10

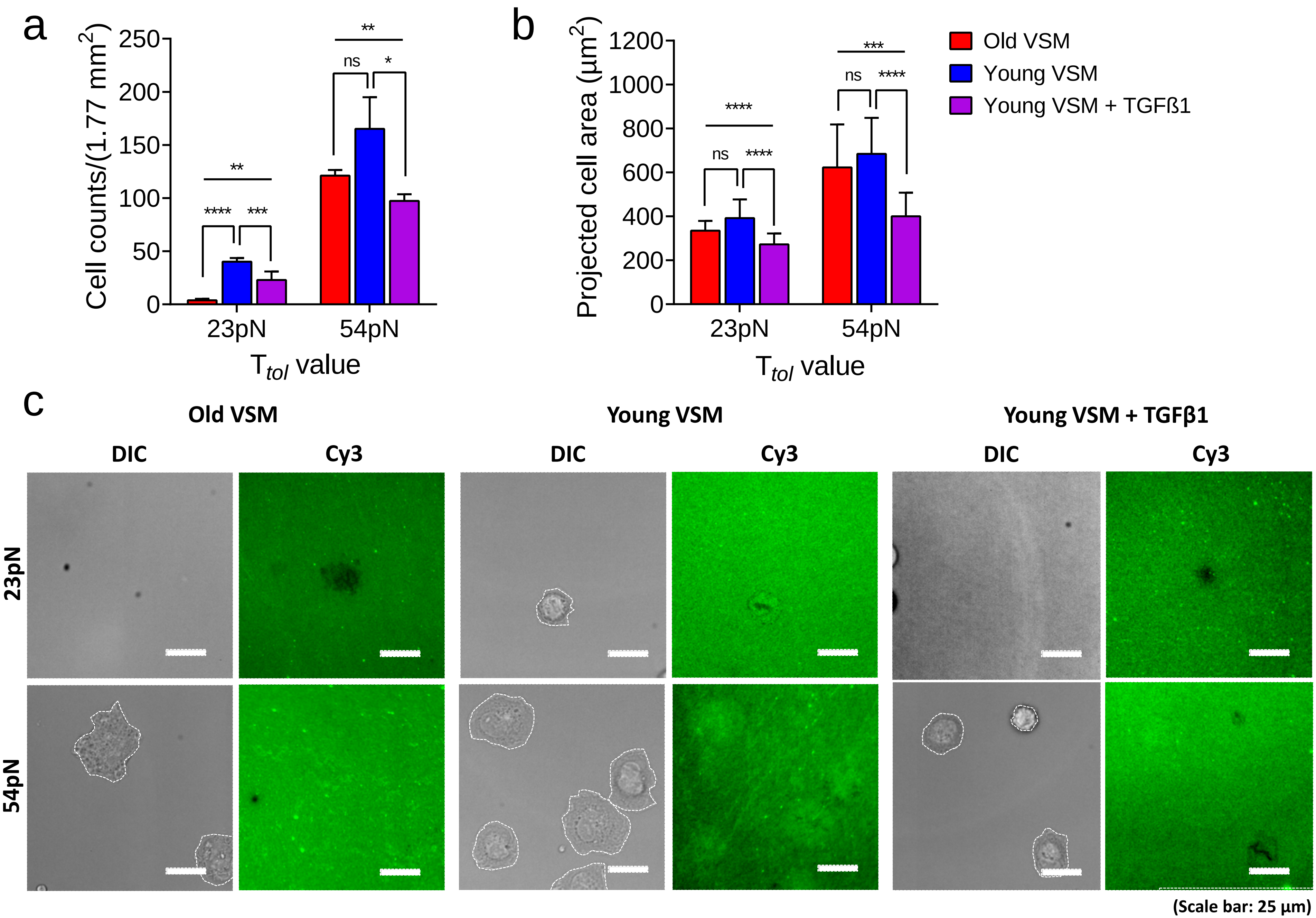
a
Cell counts/(1.77 mm^2)
250
200
150
100
50
0
**
****
***
ns
*
23pN
54pN
T_{tol} value
b
Projected cell area (μm^2)
1200
1000
800
600
400
200
0
****
***
23pN
54pN
T_{tol} value
Old VSM
Young VSM
Young VSM + TGFß1
c
Old VSM
Young VSM
Young VSM + TGFβ1
DIC
Cy3
23pN
54pN
(Scale bar: 25 μm)

Suppl. Fig. 11

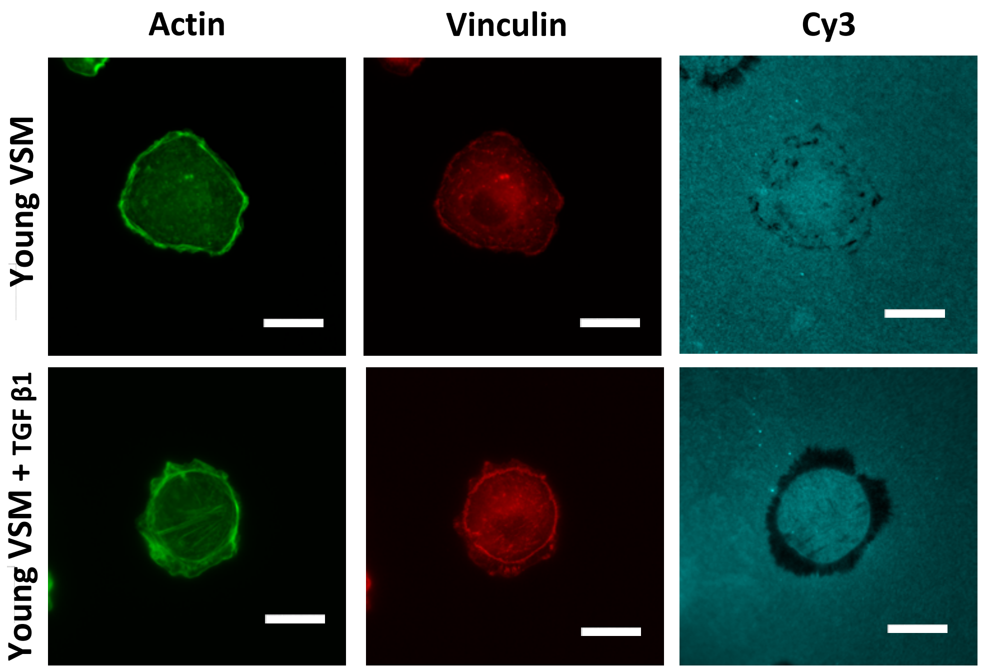

(Scale bar: 25 μm)

Suppl. Fig. 12

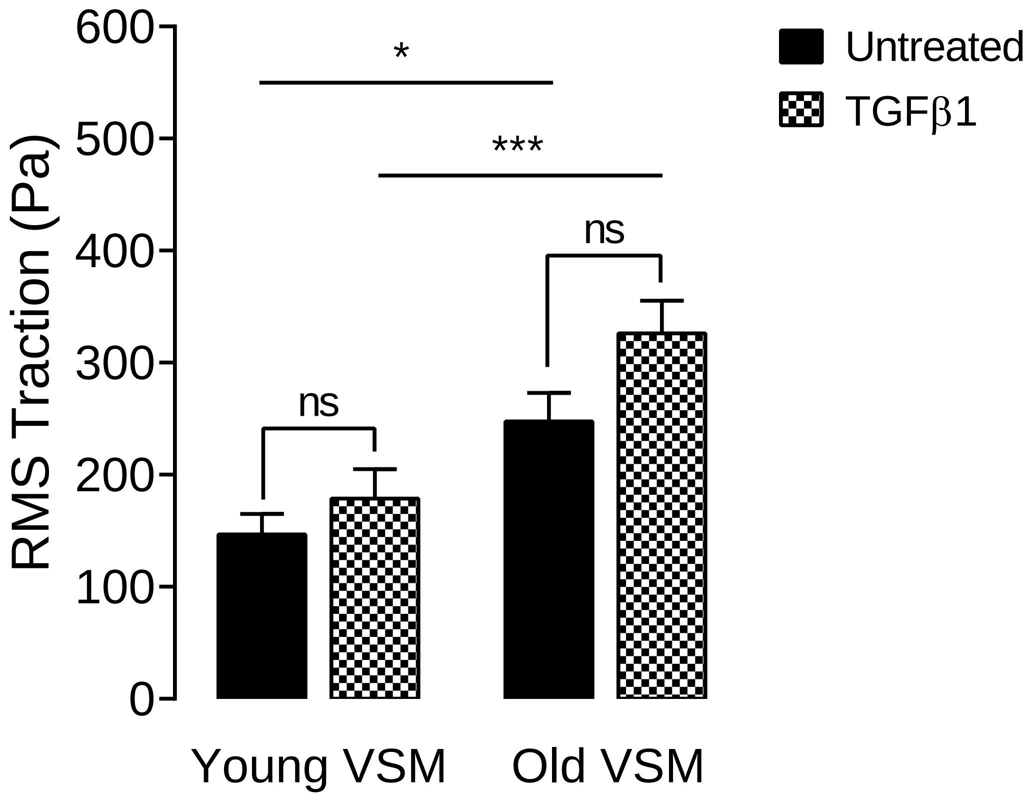

600
500
400
300
200
100
0
RMS Traction (Pa)
*
***
ns
ns
Young VSM
Old VSM
Untreated
TGFβ1
